# Experimental and Theoretical Investigations on the Supermolecular Structure of Isoliquiritigenin and 6-*O*-α-d-Maltosyl-β-cyclodextrin Inclusion Complex

**DOI:** 10.3390/ijms160817999

**Published:** 2015-08-04

**Authors:** Bin Li, Benguo Liu, Jiaqi Li, Huizhi Xiao, Junyi Wang, Guizhao Liang

**Affiliations:** 1 School of Food Science, Henan Institute of Science and Technology, Xinxiang 453003, China; E-Mails: lb55@hist.edu.cn (Bi.L.); liubenguo@hist.edu.cn (Be.L.); ljq15937322777@gmail.com (J.L.); wangjunyi21@gmail.com (J.W.); 2 Key Laboratory of Biorheological Science and Technology, Ministry of Education, School of Bioengineering, Chongqing University, Chongqing 400044, China; E-Mail: 20131902032@cqu.edu.cn

**Keywords:** isoliquiritigenin, 6-*O*-α-d-Maltosyl-β-CD, inclusion complex, ONIOM calculation

## Abstract

Isoliquiritigenin (ILTG) possesses many pharmacological properties. However, its poor solubility and stability in water hinders its wide applications. The solubility of bioactive compounds can often be enhanced through preparation and delivery of various cyclodextrin (CD) inclusion complexes. The 6-*O*-α-d-maltosyl-β-CD (G_2_-β-CD), as one of the newest developments of CDs, has high aqueous solubility and low toxicity, especially stable inclusion characteristics with bioactive compounds. In this work, we for the first time construct and characterize the supermolecular structure of ILTG/G_2_-β-CD by scanning electron microscopy (SEM), ultraviolet-visible spectroscopy (UV), Fourier transform infrared spectroscopy (FT-IR), and X-ray diffractometry (XRD). The solubility of ILTG in water at 25 °C rises from 0.003 to 0.717 mg/mL by the encapsulation with G_2_-β-CD. Our experimental observations on the presence of the ILTG/G_2_-β-CD inclusion complex are further supported by the ONIOM(our Own N-layer Integrated Orbital molecular Mechanics)-based QM/MM (Quantum Mechanics/Molecular Mechanics) calculations, typically substantiating these supermolecular characteristics, such as detailed structural assignments, preferred binding orientations, selectivity, solvent effects, interaction energies and forces of the ILTG/G_2_-β-CD inclusion complex. Our results have elucidated how ILTG interacts with G_2_-β-CD, demonstrating the primary host-guest interactions between ILTG and G_2_-β-CD, characterized by hydrogen bonds, hydrophobic interactions, electrostatic forces, and conformational effects, are favored for the formation of the ILTG/G_2_-β-CD inclusion.

## 1. Introduction

**Figure 1:**
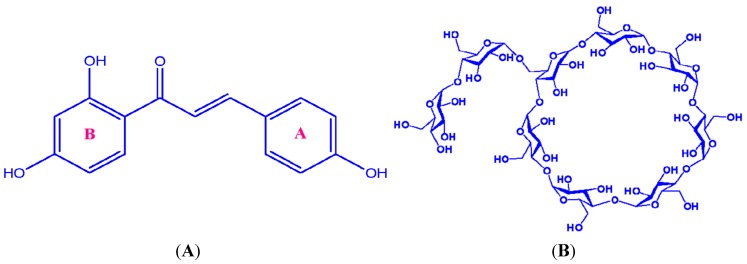
Chemical structures of (**A**) Isoliquiritigenin (ILTG) and (**B**) G_2_-β-CD.

Isoliquiritigenin (ILTG, Figure 1A), which possesses a basic structure of two benzene rings linked through an α, β-unsaturated carbonyl group, is a chalcone compound that is found in various medicinal plants such as *Glycyrrhiza uralensis* (licorice), *Allium ascalonicum*, *Sinofranchetia chinensis*, *Dalbergia odorifera*, and *Glycine max* L. [1,2]. ILTG has recently attracted wide interest due to its various pharmacological properties such as anti-inflammatory [3], antioxidant [4], anticancer [5], antiangiogenic [6], antiallergic [7], and neurological functions [2,8]. However, its poor aqueous solubility has hindered its wide applications in food, medicinal, chemical and cosmetic industry.

Cyclodextrin (CD) complexation is a widely used method for improving the aqueous solubility of bioactive molecules for enhanced delivery, stability and bioavailability [9,10]. CDs are nontoxic, cone-shaped oligosaccharides with a hydrophilic exterior and a hydrophobic cavity, making them suitable hosts for aromatic guest molecules [11,12]. Therefore, CDs have been extensively applied for numerous purposes, including enhancing solubility and bioavailability, reducing odors and haemolysis, and preventing admixture incompatibilities [13,14]. Among literally thousands of variations of CDs [15], most typical are cyclohexaamylose (α-CD), cycloheptaamylose (β-CD), and cyclooctaamylose (γ-CD) [16]. However, the low aqueous solubility of natural CDs, specially β-CDs (1.85 g 100 mL^−1^ at room temperature [17]), has restricted their wide applications. To improve their solubility, a variety of CD derivatives have been developed [18,19]. Of these hydrophilic CDs, branched CDs, in which glucose or maltooligosaccharides are linked by α-1,6 glucosidic linkages to glucose residues, are useful for the solubilization of water-insoluble or slightly soluble compounds. It should be specially mentioned natural-β-CDs have very high systemic toxicity, compared with highly water-soluble β-CD derivatives such as G2-β-CD, 2-hydroxypropyl-β-CD and sulfobutyl ether-β-CD [20], for example, they have a toxic effect on kidney as reported by Irie and Uekama [12].

For its high solubility in water, low hemolytic activity [21] and stable inclusion characteristics [22], G_2_-β-CDs (Figure 1B) can be widely used in food and pharmaceutical industry [20,23]. However, to the best of our knowledge, there is no report about the interaction mechanisms of G_2_-β-CD with guest molecules. Various experimental approaches, such as UV, FT-IR, XRD and NMR [24], have been employed to characterize the supermolecular structures of bioactive compounds with CDs (or their variations), however, it is often difficult, or too time consuming, to obtain detailed host-guest interaction mechanisms, or some means usually provide only very indirect information or about inclusion modes and geometries [25,26]. Fortunately, an increasing number of theoretical methods, such as semiempirical methods, density functional theory (DFT) and molecular dynamics (MD) simulation, have currently been used to describe complexation energetics of supermolecular systems [27]. However, the accuracy of semiempirical calculations (such as AM1 and PM3) is relative low, and the DFT methods are prohibitively expensive to carry out studies on such large systems, and for MD methods [9,28], it is difficult to construct the force fields for CD variations and guest molecules. To overcome these drawbacks, a hybrid ONIOM (our Own N-layer Integrated Orbital molecular Mechanics) method [29] was developed to perform a complex geometry optimization. In this method, it simultaneously treats different parts of a system with good accuracy and lower computational cost compared to pure DFT methods [30]. For molecule/CD systems, under many circumstances, the CD only provides an environment effect, and we are more interested in the specific properties of the guest molecules. Based on these considerations, the ONIOM-based method appears to be promising to study molecule/CD chemistry.

In this work, we focus on characterization of the host-guest interactions in the ILTG/G_2_-β-CD complex by a combination of experimental and computational approaches, such as SEM, UV, FT-IR, XRD, and ONIOM calculations. Our experimental and theoretical results have mutually supported to clarify how ILTG interacts with G_2_-β-CD.

## 2. Results and Discussion

### 2.1. Enhanced Solubility of Isoliquiritigenin (ILTG) by the Inclusion with G_2_-β-CD

The ILTG molecule has a very low solubility, thus we employed one promising method, the encapsulation by G_2_-β-CD, to improve its solubility. The CD systems have very low or no toxicity, and are easily accessible and possess a high biocompatibility. Moreover, complexation with CDs appears to offer a number of advantages [31], for example, it does not alter the chemical structures of the studied compounds, and they have the ability both to enhance drug delivery through biological membranes [13] and to control the release rate of drugs [32].

**Figure 2:**
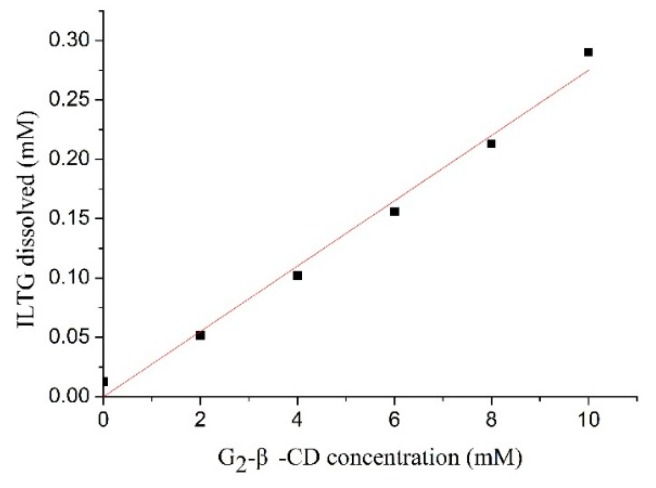
Phase–solubility diagram of the complexes formed by ILTG and G_2_-β-CD.

As shown in Figure 2, the phase-solubility plot of the complex of ILTG/G_2_-β-CD displays a typical AL type diagram (*i.e.*, linear increases in ILTG solubility with increasing G_2_-β-CD concentration), indicating 1:1 molecular complex [10]. We compared the solubility of ILTG before and after the formation of the inclusion complex by the UV method. It is found that the solubility of ILTG was only 0.003 mg/mL in deionized water at 25 °C, while the aqueous solution of G_2_-β-CD enhanced the solubility of ILTG up to 0.717 mg/mL. Therefore, the next work was to determine if the ILTG/CD inclusion complex was formed as well as the interaction characteristics of ILTG with G_2_-β-CD, thereby to explain why the solubility of ILTG was significantly improved by G_2_-β-CD.

### 2.2. SEM Analysis

**Figure 3:**
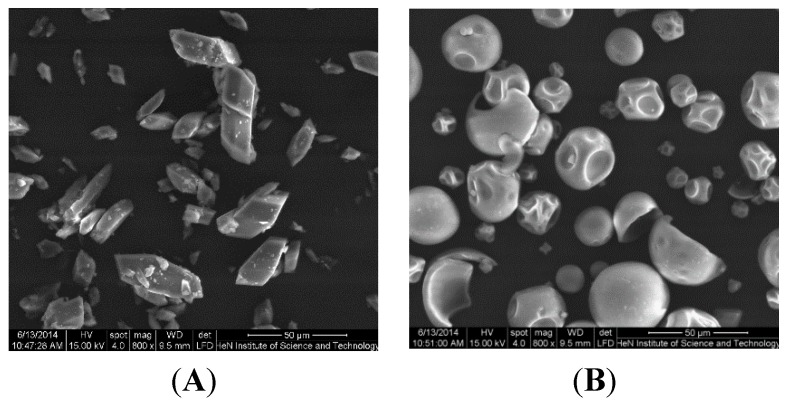

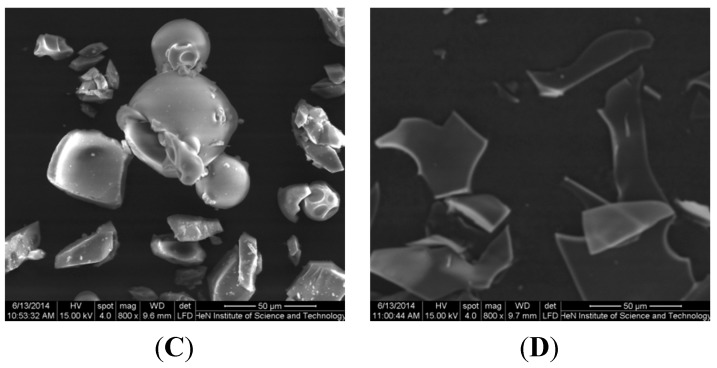
Scanning electron micrographs of (**A**) ILTG, (**B**) G_2_-β-CD, (**C**) the ILTG/G_2_-β-CD physical mixture, and (**D**) the ILTG/G_2_-β-CD inclusion complex.

The first evidence of the formation of the ILTG/G_2_-β-CD inclusion complex was experimentally obtained by comparing the scanning electron micrographs of G_2_-β-CD, ILTG, and their physical mixture and inclusion complex displayed in Figure 2. It shows that ILTG exists in rectangular crystals (Figure 3A) while G_2_-β-CD appears as amorphous spheres (Figure 3B). In the electron micrograph of the physical mixture of ILTG with G_2_-β-CD, both the characteristic crystals of ILTG and the amorphous spheres of G_2_-β-CD are observed (Figure 3C). In contrast, the inclusion complex appears as irregular particles in which the original morphology of both components disappears and tiny aggregates of amorphous pieces of irregular sizes are present (Figure 3D). These images demonstrated that when the powders of ILTG and G_2_-β-CD were simply mixed together, they could form no close association and continue to exist in their original individual forms, whereas when the solutions of the two compounds were freeze-dried, they could form a close association, probably in the form of inclusion complex, in which ILTG no longer existed in the crystal state.

### 2.3. UV Analysis

**Figure 4:**
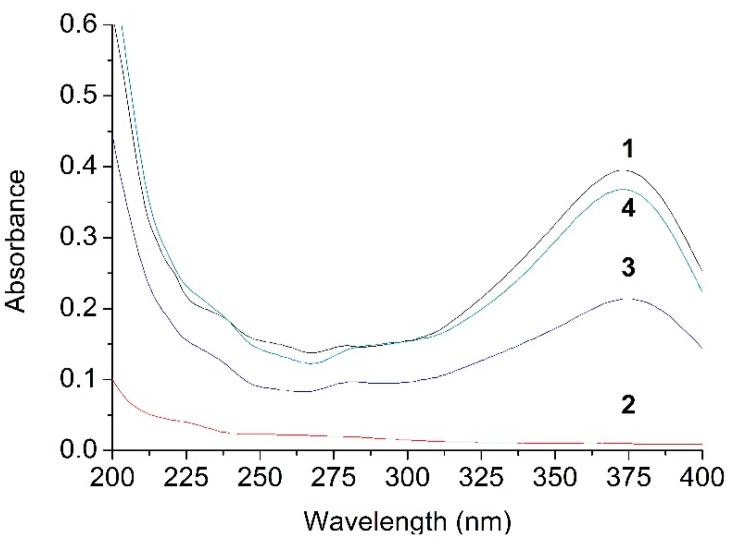
UV spectra of (1) ILTG (373 nm), (2) G2-β-CD, (3) the ILTG/G2-β-CD physical mixture (373 nm), and (4) the ILTG/G2-β-CD inclusion complex (373 nm).

Figure 4 illustrates that the UV absorbance of G_2_-β-CD is very low with no appreciable peak due to the absence of π-electrons or non-bonding electrons. It is important to notice that the UV spectra of their physical mixture (373 nm) and complex (373 nm) are the same as that of ILTG (373 nm), suggesting there was no covalent interaction between G_2_-β-CD and ILTG.

### 2.4. FT-IR Analysis

**Figure 5:**
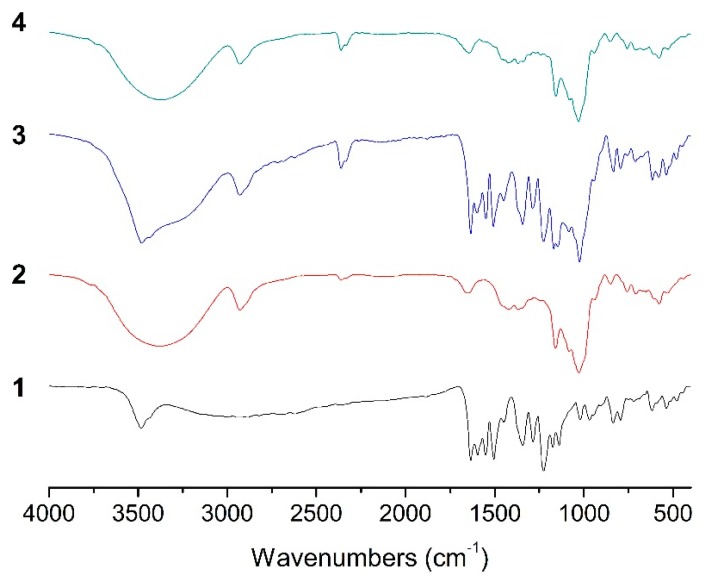
IR spectra of (1) ILTG, (2) G2-β-CD, (3) the ILTG/G2-β-CD physical mixture, and (4) the ILTG/G2-β-CD inclusion complex.

The variation of the shape, shift, and intensity of the IR absorption peaks of the guest or host molecule can provide enough information for the occurrence of the inclusion. The FT-IR spectra of ILTG consists of the prominent absorption bands at 3482 cm^−1^ (for O–H stretching vibration), 1634 cm^−1^ (for C=O group), 1596, 1551, 1505 and 1449 cm^−1^ (for aromatic nucleus). The FT-IR spectrum of G_2_-β-CD shows the main absorption bands at 3376 cm^−1^ (for O–H stretching vibration), 2928 cm^−1^ (for C–H stretching vibration), 1653 (O-H bending vibration), 1159 and 1027 cm^−1^ (for C–H and C–O stretching vibration) (Figure 5). The FT-IR spectrum of the physical mixture reveals a spectral addition effect and is essentially a combination of the spectra of the two molecules. It is also observed the shape of the FT-IR spectrum of the ILTG/G_2_-β-CD inclusion complex is very close to that of G_2_-β-CD; furthermore, some small peaks ranging from 400 to 1600 cm^−1^ for ILTG are overshadowed by the corresponding ones of G_2_-β-CD, with no new absorption peaks observed, suggesting no new chemical bond was formed in the inclusion process of G_2_-β-CD and ILTG.

### 2.5. X-ray Powder Diffraction Pattern Analysis

**Figure 6:**
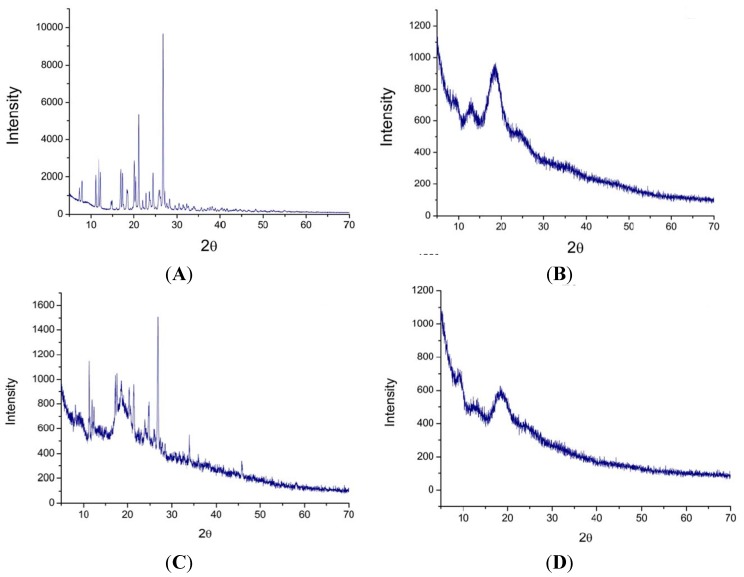
XRD patterns of (**A**) ILTG, (**B**) G2-β-CD, (**C**) the ILTG/G2-β-CD physical mixture, and (**D**) the ILTG/G2-β-CD inclusion complex.

The complexation of ILTG with G_2_-β-CD was further investigated by XRD. Many intense and sharp peaks can prove the crystalline nature of the compound [33], thus as can be seen from the XRD patterns of ILTG (Figure 6A), there are strong crystallinity peaks at 2θ of 7.34°, 7.92°, 11.11°, 11.75°, 12.16°, 16.89°, 17.27°, 18.31°, 20.11°, 20.44°, 21.16°, 22.81°, 24.45° and 26.64°. Figure 6B displays the XRD patterns of G_2_-β-CD have a broad peak in the range of 16°–22°, consistent with its amorphous character. For their physical mixture, the diffraction pattern is simply the superposition of the two patterns of the crystalline ILTG and the amorphous G_2_-β-CD (Figure 6C), demonstrating no inclusion was formed between two compounds and they still retained their original physical characteristics. However, the inclusion complex of ILTG and G_2_-β-CD reveals a large amorphous peak (16°–22°, Figure 6D), which is similar to that of G_2_-β-CD and exhibits none of the crystallinity peaks of ILTG. Accordingly, the experimental XRD results demonstrated most of ILTG has been dispersed in G_2_-β-CD matrix and lost its crystallinity.

### 2.6. Binding Orientations of G_2_-β-CD with ILTG

The evidences of the presence as well as its characteristics of the ILTG/G_2_-β-CD supermolecular complex were further confirmed by theoretical simulations. It is time-and resource-consuming to optimize the ILTG/G_2_-β-CD complex in water by ONIOM (B3LYP/6-31+G(d):PM3) calculations; therefore, we first carried out the optimization of binding orientations of ILTG with G_2_-β-CD in the gas phase.

**Figure 7:**
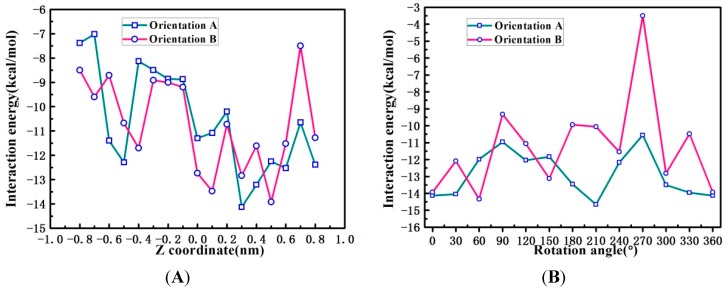
Interaction energies of the inclusion complexation of ILTG into the cavity of G_2_-β-CD at different (**A**) *Z* positions and (**B**) rotation angles in the gas phase by ONIOM (B3LYP/6-31+G(d):PM3) calculations.

The curves for interaction energy of two kinds of models in the gas phase (Figure 7) show the lowest $∆E_{Int}$ in all the traversing models is −14.13 kcal/mol for orientation A while −13.92 kcal/mol for orientation B. On basis of the most stable orientation in the traversing models, we then obtained the stable orientations in all the rotating models with the lowest energies of −14.66 kcal/mol for orientation A and −14.33 kcal/mol for orientation B. As we know, the lower $∆E_{Int}$, the stronger host-guest interaction. Therefore, the $∆E_{Int}$ above displayed that ILTG preferably passed through from the wide rim of G_2_-β-CD and finally formed a stable inclusion complex with G_2_-β-CD (The optimized complex structure with PDB format is displayed in Supplementary File 1). Specially, as shown in Figure 6, ILTG completely inserts inside the intramolecular hydrophobic cavity of G_2_-β-CD in orientation A, whereas G_2_-β-CD does not form a good inclusion complex with ILTG by means of orientation B in which the phenyl moiety of ILTG lies at the outside of the G_2_-β-CD cavity.

### 2.7. Solvent Effects on Binding Selectivity of G_2_-β-CD with ILTG

The ILTG/G_2_-β-CD inclusion was not prepared in the gas phase but in water, thus it is not reliable enough to explore the gas-phase binding orientations of G_2_-β-CD with ILTG. This is because in the gas phase there is no solvent to interact with the polar groups such as the hydroxyls, the intra- and intermolecular hydrogen bonds with which the negative charge is efficiently distributed, which are believed to constitute the primary interactions in the molecule/CD system. Therefore, based on the most stable inclusion obtained from orientation A optimized in the gas phase, we further optimized the most potentially stable ILTG/G_2_-β-CD complex in water at the ONIOM (B3LYP/6-31+G(d):PM3) level of theory, thereby to explore solvation effects on the inclusion structure.

**Figure 8:**
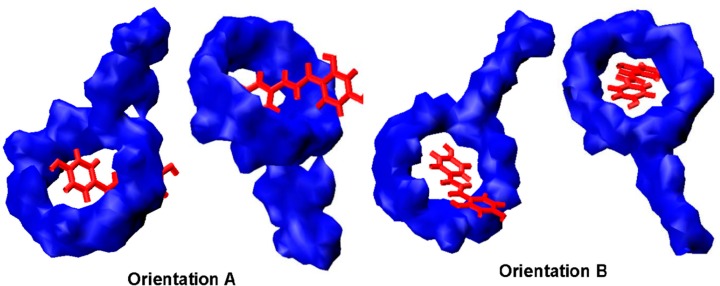
Complexation snaps of the ILTG/G_2_-β-CD complex in the gas phase by ONIOM (B3LYP/6-31+G(d):PM3) calculations. The left two snaps denote the conformations obtained from Orientation A, while the right two snaps denote the conformations obtained from Orientation B.

The calculated binding orientations of ILTG with G_2_-β-CD show there is no significant difference between the conformations optimized in the gas phase and water (Figure 8), however, the $∆E_{Int}$ (−10.97 kcal/mol) in water is distinctly higher than the gas-phase $∆E_{Int}$ (−14.66 kcal/mol), suggesting the stability of the ILTG/G_2_-β-CD inclusion conformation has been affected by solvation effects. This finding can be largely interpreted by the following reasons: ILTG is a non-polar molecule, and G_2_-β-CD has a hydrophobic inner cavity. Thus, once they come into the cavity of G_2_-β-CD, polar water molecules can affect the interactions of G_2_-β-CD with ILTG; in other words, relative to the gas phase, from a quantum-chemical point of view, water molecules are not conducive to the formation of the ILTG/G_2_-β-CD inclusion, which is consistent with the characteristics of high-energy water as described in a recent review [34].

### 2.8. Calculated UV Spectra in Water

**Figure 9:**
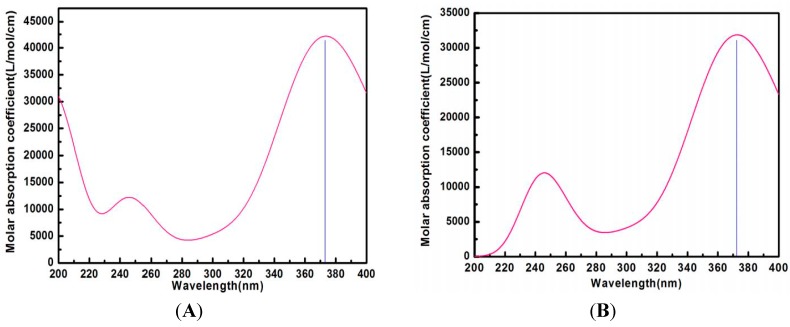
Calculated UV spectra by the B3LYP/6-31+G(d) level of theory. (**A**) ILTG (374 nm); and (**B**) the ILTG/G_2_-β-CD inclusion complex (373 nm).

To further validate the presence of the ILTG/G_2_-β-CD supermolecular structure, we explored the calculated UV spectra characteristics for ILTG and ILTG/G_2_-β-CD in water using the IEF-PCM (Integral Equation Formalism for the Polarizable Continuum Model) [35]. For comparison purposes, the UV spectra of both ILTG and the complex was calculated by the time-dependent density functional theory (TDDFT) method with the B3LYP functional and 6-31+G(d) basis set. It is important to note there is only a 1 nm difference between the experimental (373 nm) and calculated (374 nm) maximum absorption peaks for ILTG (Figure 9), while the simulated UV spectra (373 nm, Figure 9) of the complex are consistent with its experimental spectra (373 nm, Figure 4). Accordingly, the high-precision calculated UV spectra enlightens us that the UV spectral characteristics of ILTG were not altered due to the presence of G_2_-β-CD, further supporting our previous conclusion that no new chemical bond was formed between ILTG and G_2_-β-CD. In the ILTG/G_2_-β-CD inclusion complex, the non-covalent interactions between ILTG and G_2_-β-CD such as hydrogen bonds, hydrophobic, and electrostatic interactions will lower the absorbance of the corresponding atoms. Based on this consideration, we noticed the calculated molar absorption coefficient (L/mol/cm) for the complex experiences a significant reduction relative to that of ILTG (Figure 9). In conclusion, our calculated UV spectra have exhibited a good agreement with the experimental UV spectra; furthermore, the use of the TDDFT-based technique mixed the B3LYP functional and 6-31+G(d) basis set appears to be a satisfactory methodology to describe the UV spectra characteristics for the ILTG/G_2_-β-CD complex in water using the IEF-PCM model.

### 2.9. Energies and Forces between ILTG and G_2_-β-CD in Water

There are many oxygen atoms that serve as Lewis base sites and hydroxyl groups acting as Lewis acid contributors [27], therefore, we investigated what roles H-bonds play in the inclusion process of G_2_-β-CD with ILTG by NBO analysis [36]. In NBO analysis, a stabilization energy (E^(2)^) is used to characterize the hydrogen bond interactions between occupied Lewis-type NBO orbitals and formally unoccupied non-Lewis NBO orbitals, which reflects the delocalization trend of electrons from the bonding (BD) or nonbonding orbitals (LP) to the antibonding orbitals (BD *).

**Figure 10:**
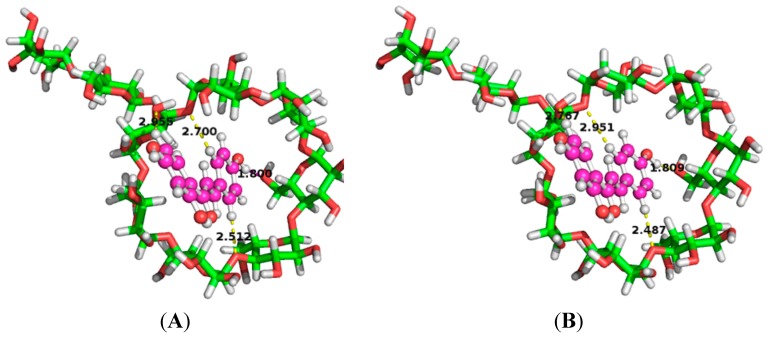
Binding conformations ILTG with G_2_-β-CD by ONIOM (B3LYP/6-31+G(d):PM3) calculations in (**A**) water and (**B**) gas phase. The dotted lines denote intermolecular hydrogen bonds.

It is reported, in general, the E^(2)^ value is larger than 2.00 kcal/mol for strong H-bond interactions and from 0.50 to 2.00 kcal/mol for weak H-bond interactions [28]. As shown in Figure 10, there are four intermolecular H-bonds in the inclusion complex described by dashed lines. The H···O distances and angles at H atom in these H-bonds (C–H···O or O–H···O) range from 1.80 to 2.96 Å and from 122.91° to 159.68°, respectively, which just fall in the reported scopes (less than 3.2 Å and greater than 90°) [24]. Distance and angles and NBO donor-acceptor interaction energies E^(2)^ of the four H-bonds (Table 1) show the largest E^(2)^ values are 2.93 and 2.95 kcal/mol in the gas phase and water, respectively, suggesting there are one strong intermolecular electron donor-acceptor orbital interaction corresponding to the O–H···O contact. Additionally, there are two weak H-bonds and one very weak H-bond which involved the intermolecular C–H···O contact.

As can be seen from the direction of these H-bonds, the electron is transferred from G_2_-β-CD to ILTG, in which electrons are provided by the oxygen atom in the OH group for the H-bond with the highest E^(2)^, while by the oxygen atoms in the glycosidic bond for the other 3 weak H-bonds. The host-guest charge transfer between ILTG and G_2_-β-CD suggests the host molecule loses electrons while the guest molecule acquires electrons in the ILTG/G_2_-β-CD inclusion complex (Table 2), which is consistent with the charge transfer direction in the four H-bonds analyzed above. Moreover, the charge transfer between ILTG and G_2_-β-CD in water is more significant than that in the gas phase. In addition, as described above, the phenyl moiety of ILTG inserted into the hydrophobic cavity of G_2_-β-CD, demonstrating there are strong hydrophobic forces between ILTG and G_2_-β-CD.

**Table 1:** Distance and angles and NBO donor-acceptor interaction energies E^(2)^ for intermolecular hydrogen bonds in ILTG/G_2_-β-CD complexes by ONIOM (B3LYP/6-31+G(d):PM3) calculations.

Medium	Donor	Acceptor	Distance (Å)	Angle (°)	E^(2)^ (kcal/mol)
Water	LP(1) O55	BD * (1) C201-H214	2.51	159.68	1.70
	LP(1) O62	BD * (1) H211-O217	1.80	155.47	2.95
	LP(2) O74	BD * (1) C195-H207	2.96	125.80	0.31
	LP(1) O75	BD * (1) C200-H215	2.70	140.55	0.77
Gas phase	LP(1) O55	BD * (1) C201-H214	2.49	156.88	1.75
	LP(1) O62	BD * (1) H211-O217	1.81	156.41	3.00
	LP(2) O74	BD * (1) C195-H207	2.77	145.84	0.49
	LP(1) O75	BD * (1) C200-H215	2.95	122.91	0.27

The label number of atoms of the complex could be seen in the optimized 2D planar structure in supplementary material (SFile 2). BD denotes σ bonding orbital; BD * denotes σ * antibonding orbital; LP denotes valence lone pair. For BD and BD *, (1) denotes σ orbital, (2) denotes π orbital. For LP, (1) and (2) denote the first and the second lone pair electron, respectively. Distance denotes the H···O distance of C–H···O or O–H···O; Angle denotes the angel of C–H···O or O–H···O.

**Table 2:** Dipole moment and charge of ILTG and G_2_-β-CD by ONIOM (B3LYP/6-31+G(d):PM3) calculations.

Item	Water	Gas Phase
Dipole moment (Debye)	3.233	2.586
Charge of ILTG (e)	−0.053	−0.046
Charge of G_2_-β-CD (e)	0.053	0.046

### 2.10. Adaptation Evidence of ILTG Induced by G_2_-β-CD

We shed light on adaptation alternations of ILTG by forming the inclusion with G_2_-β-CD. Energies of the highest occupied molecular orbital (HOMO) and lowest unoccupied molecular orbital (LUMO) are very popular quantum-chemical descriptors [13]. The LUMO as an electron acceptor represents the ability to acquire an electron and HOMO represents the ability to donate electron. The energy difference between HOMO and LUMO (GAP_HOMO-LUMO_) manifests the stabilization of the inclusion complex [13]. A lower GAP_HOMO-LUMO_ means that the inclusion complex is more stable [37]. The calculated results of molecular orbital energies (Table 3) indicate the GAP_HOMO-LUMO_ values in the complex in the gas phase and water are −138.78 and −135.88 kcal/mol, respectively, demonstrating the ILTG/G_2_-β-CD inclusion complex in the gas phase is more stable, which exhibits the same tendency with the $∆E_{Int}$ analysis.

**Table 3:** Quantum-chemical descriptors of ILTG before and after forming the inclusion with G_2_-β-CD at the B3LYP/6-31+G(d) level of theory.

Parameters	Water		Gas Phase	
	Free	Complex	Free	Complex
$E_{Def}^{ILTG}$ (kcal/mol)	0.75		0.71	
Dipole moment (Debye)	3.597	3.680	2.697	2.830
HOMO (kcal/mol)	−166.33	−166.20	−165.29	−165.18
LUMO (kcal/mol)	−30.92	−30.32	−26.68	−26.40
GAP_HOMO-LUMO_ (kcal/mol)	−135.41	−135.88	−138.61	−138.78
Overall surface area (Å^2^)	282.40	282.96	283.03	283.49
Positive surface area (Å^2^)	117.29	117.70	118.97	119.05
Negative surface area (Å^2^)	165.11	165.26	164.06	164.43

The total dipole moment reflects the global polarity of a molecule. It is found dipole moments of ILTG from the optimized complex are larger than those of the individual optimized ILTG. Hence, after forming the inclusion with G_2_-β-CD, the guest molecule exhibits a significant improvement in its global polarity. It is interesting to notice that the $E_{Def}^{ILTG}$ values of ILTG in the gas phase and water are all positive; particularly, $E_{Def}^{ILTG}$ in water (0.75 kcal/mol) is larger than that in the gas phase (0.71 kcal/mol), demonstrating there are more distinct driving forces in promoting ILTG to form the complexation with G_2_-β-CD in water. In addition, surface characteristics reveal ILTG has experienced distinct improvements in overall surface area, positive surface area and negative surface area (Table 3), indicating the conformation of ILTG has been altered in the inclusion process with G_2_-β-CD to favorably form the most stable inclusion complex.

## 3. Experimental Section

### 3.1. Chemicals

ILTG (98.0%) and G_2_-β-CD (98.0%, MW1459.27) was purchased from Aladdin (Shanghai, China). Other chemicals were of analytical grade unless stated otherwise.

### 3.2. Phase Solubility Study

The phase solubility studies were conducted according to the previous method [10]. An excess amount of ILTG (30 mg) was added to 5 mL of G_2_-β-CD solutions with the concentration ranging from 0 to 10 mM in test tubes. The tubes were shaken in the water bath for 72 h at 35 °C. Then, the suspensions were filtered through 0.45 μm membrane filters to remove undissolved ILTG. The concentration of ILTG in the filtrate was determined by measuring its absorbance with a TU-1810PC UV spectrophotometer (Purkinje, Beijing, China) at 373 nm and comparing it with a standard curve of pure ILTG. The phase solubility profiles of ILTG were obtained by plotting the solubility of ILTG *vs.* the concentration of G_2_-β-CD.

### 3.3. Preparation of the Inclusion Complex and Physical Mixture of ILTG and G_2_-β-CD

ILTG (0.128 g, 0.5 mmol) and G_2_-β-CD (0.730 g, 0.5 mmol) were mixed in 25 mL deionized water, stirred for 72 h at 35 °C, and filtered through a 0.45 μm membrane filter to remove undissolved material. The filtrate was freeze-dried (α 1–2, Christ, Germany) and the resultant powdery material was weighed and collected as the inclusion complex of ILTG and G_2_-β-CD. ILTG (0.128 g) and G_2_-β-CD (0.730 g) were mixed thoroughly in a small beaker at room temperature. The obtained product was collected as the physical mixture of ILTG and G_2_-β-CD.

### 3.4. Scanning Electron Microscopy

SEM analysis was performed with a Quanta 200 environmental scanning electron microscope (FEI, Hillsboro, OR, USA). The samples were evenly distributed on SEM specimen stubs with double adhesive tape. The micrographs were obtained with an accelerating potential of 15 kV under low vacuum.

### 3.5. Ultraviolet-Visible Spectroscopy

The UV spectra were recorded for G_2_-β-CD, ILTG as well as their physical mixture and inclusion complex on a model TU1810 scanning UV spectrophotometer (Beijing Purkinje General Instrument Co., Ltd., Beijing, China). Each sample was dissolved in deionized water at ambient temperature (25 ± 1 °C). The absorbance of each solution was scanned in the wavelength range 220−400 nm to obtain the UV spectra. The solubility of ILTG and its complex in deionized water was also determined by UV method as following: the excess of ILTG and its complex was added to 5 mL water at 25 °C. The obtained solutions were agitated for 24 h, and then filtered. The supernatant was diluted with ethanol, which was analyzed by spectrophotometry at 373 nm to determine the concentration of dissolved ILTG.

### 3.6. Fourier Transform Infrared Spectroscopy

The FT-IR spectra of G_2_-β-CD, ILTG as well as their physical mixture and inclusion complex were collected between 4000 and 400 cm^−1^ on a Tensor 27 infrared spectrophotometer (Bruker, Billerica, MA, USA) with 256 scans at a resolution of 4 cm^−1^ by the KBr method. The data were recorded and processed by Opus software supplied with the instrument.

### 3.7. X-ray Diffractometry

For XRD, monochromatic Cu Kα radiation (wavelength = 1.54056 Å) was produced by a D8 Advance X-ray diffractometer (Bruker, Billerica, MA, USA). The powdery samples were packed tightly in a rectangular aluminum cell prior to exposure to the X-ray beam. The scanning regions of the diffraction angle, 2θ, were 5°−80°, and radiation was detected with a proportional detector.

### 3.8. ONIOM Host-Guest Simulations

**Figure 11:**
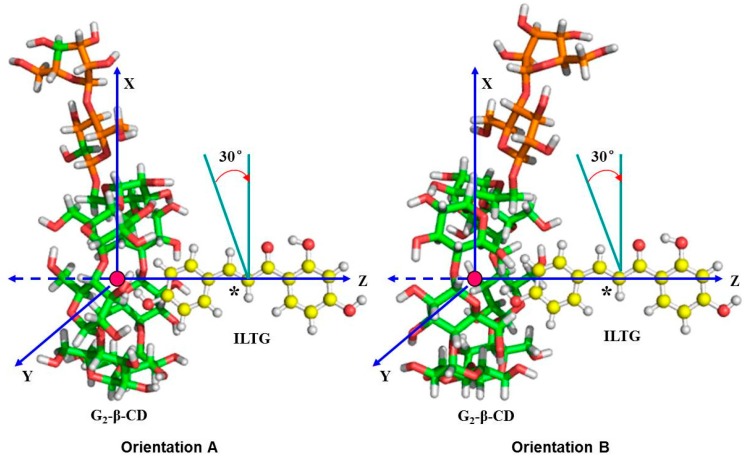
Binding orientations used to define the inclusion processes of -β-CD with ILTG. ILTG is displayed in yellow by ball and stick while G_2_-β-CD is displayed in a combination of green (β-CD) and orange (G_2_) by stick. Orientation A denotes that ILTG enters into the cavity from the wide rim of G_2_-β-CD, while Orientation B denotes that ILTG enters into the cavity from the narrow rim of G_2_-β-CD. The position of ILTG is determined by the *Z*-coordinate of the labeled carbon atom (*) from the center of the glycosidic oxygen atoms.

We constructed two kinds of models, traversing and rotating models, to search the most stable supermolecular structure of the ILTG/G_2_-β-CD complex in which ILTG formed the inclusion with G_2_-β-CD in molar proportion 1:1 (one ILTG and one G_2_-β-CD). The glycosidic oxygen atoms of G_2_-β-CD were placed onto the *XY* plane and their center was defined as the center of the coordination system. Then, G_2_-β-CD was maintained in this position while ILTG was introduced along *Z*-axis into the cavity of G_2_-β-CD. The labeled carbon atom of ILTG was initially located at +0.8 nm on *Z*-axis and was moved into the G_2_-β-CD cavity along *Z*-axis in 0.1 nm step until −0.8 nm (Figure 11). Two possible orientations, Orientation A in which the A ring of ILTG pointed toward the wider rim of G_2_-β-CD and Orientation B in which the A ring of ILTG pointed toward the narrower rim of G_2_-β-CD, were considered. To find an even more stable structure of the complex, the structures obtained from the most stable traversing models were treated as initial ones for rotating models, in which ILTG was rotated around Z-axis by 30° from 0° to 360°.

All generated geometries at each step were completely optimized in all internal degrees of freedom using a two layered hybrid ONIOM method [29], in which the inner layer ( ILTG) was treated at the B3LYP [38]/6-31+G(d) level of theory, while the outer layer (G_2_-β-CD) was treated by the semiempirical PM3 method. For all the optimized models, frequency calculations were subsequently performed, with no imaginary frequency results. Thereafter, the global minimum points were recognized, and the ultimate conformation and a serial of quantum-chemical parameters, including various energies, dipole moments, surface characteristics, *etc.*, were obtained.

To elucidate solvent effects on the conformational equilibrium of the inclusion, we optimized the stable gas-phase ILTG/G_2_-β-CD superstructure in water by an integral equation formalism polarized continuum model IEF-PCM method [35] by the ONIOM(B3LYP/6-31+G(d):PM3) level of theory. All DFT and IEF-PCM calculations were performed using Gaussian 09 [39].

### 3.9. ONIOM Interaction Calculations

In the ONIOM terminology of Morokuma and co-workers [29], the full system is called “real” and is treated with a low level of theory. The inner layer is termed “model” and is treated with both the low level of theory and a high level of theory. Total energy *E*^ONIOM^ is then expressed by the equation below:

(1) $$E^{\text{ONIOM}}=E\text{(high, model)}+E\text{(low, real)}-E\text{(low, model)}$$

The host-guest interaction energies were corrected for basis set superposition errors (BSSEs) as calculated with the counterpoise method [40]. Interaction energy ($∆E_{Int}$) was characterized by the energetic difference between the complexed form and isolated species expressed as Equation (2) ($E_{Comp}^{CD}$ and $E_{Comp}^{ILTG}$ corresponds to the energy of G_2_-β-CD and ILTG in the complex, respectively). The deformation energy of ILTG ($E_{Def}^{ILTG}$, Equation (3)) was used to characterize the difference between the optimized geometry energy ($E_{Free}^{ILTG}$) and single point energy ($E_{Comp}^{ILTG}$) in their complex geometry.

(2) $$∆E_{Int}=E^{ONIOM}-E_{Comp}^{CD}-E_{Comp}^{ILTG}$$

(3) $$E_{Def}^{ILTG}=E_{Comp}^{ILTG}-E_{Free}^{ILTG}$$

Natural bond orbital (NBO) calculations were used to analyze the intermolecular interactions. The electronic wave functions are interpreted in term of a set of occupied Lewis orbitals and a set of non-Lewis localized orbitals. The second order perturbation theory was used to estimate the electronic delocalization interactions which can quantitatively be described by the stabilization energy (*E*^(2)^) expressed as Equation (4) [36]:

(4) $$E^{\left(2\right)}=-n_{\text{σ}}\frac{\langle \text{σ}\left|F\right|\text{σ}\rangle }{{\text{ε}}_{{\text{σ}}^{\text{*}}}-{\text{ε}}_{\text{σ}}}=-n_{\text{σ}}\frac{F_{i,j}^{2}}{∆E}=-n_{\text{σ}}\frac{F_{i,j}^{2}}{E_{j}-E_{i}}$$

where 〈σ|F|σ〉 or $F_{i,j}^{2}$ is the Fock matrix element between the *i* and *j* NBO orbitals, and $F_{i,j}$ denotes the off-diagonal NBO Fock matrix element. The ${\text{ε}}_{{\text{σ}}^{\text{*}}}$ and ${\text{ε}}_{\text{σ}}$ denote the energies of σ^*^ and σ NBO orbitals, and *n*_σ_ is the population of the donor σ orbital. The *E_i_* and *E_j_* denote diagonal elements (orbital energies).

## 4. Conclusions

The solubility of ILTG in water is enhanced by forming the inclusion complex with G_2_-β-CD. We characterize the supermolecular structure of the ILTG/G_2_-β-CD inclusion complex as experimentally evidenced by SEM, UV, FT-IR, and XRD. Experimental results demonstrate the formation of the supermolecular complex in which ILTG is entrapped within the cavity of G_2_-β-CD. Theoretical simulations substantiate the experimental data by evidencing the stability of the inclusion of ILTG in the G_2_-β-CD cavity as well as explaining the related changes of physicochemical property of ILTG. The detailed structural alignment, preferred binding orientations, selectivity, interaction energies of the G_2_-β-CD with ILTG are typically computed to elucidate how G_2_-β-CD interacts with ILTG by high-precision ONIOM calculations at the B3LYP/6-31+G(d):PM3 level of theory, together with the calculation of UV spectra by the B3LYP/6-31+G(d) level of theory. This work performs for the first time the elucidation on the ILTG/G_2_-β-CD supermolecular complex, providing valuable reference for employing G_2_-β-CD or other variations of CDs to improve the solubility or acquire specific properties of the investigated compounds in pharmaceutical, chemical, food, and nanoscience applications.

## Supplementary Files

Supplementary File 1
