# Supplementary figures and images for: Experimental and Theoretical Investigations on the Supermolecular Structure of Isoliquiritigenin and 6-O-α-d-Maltosyl-β-cyclodextrin Inclusion Complex
Source: Int J Mol Sci. 2015 Aug 4;16(8):17999–8017. doi: 10.3390/ijms160817999 (PMC4581232; doi:10.3390/ijms160817999)

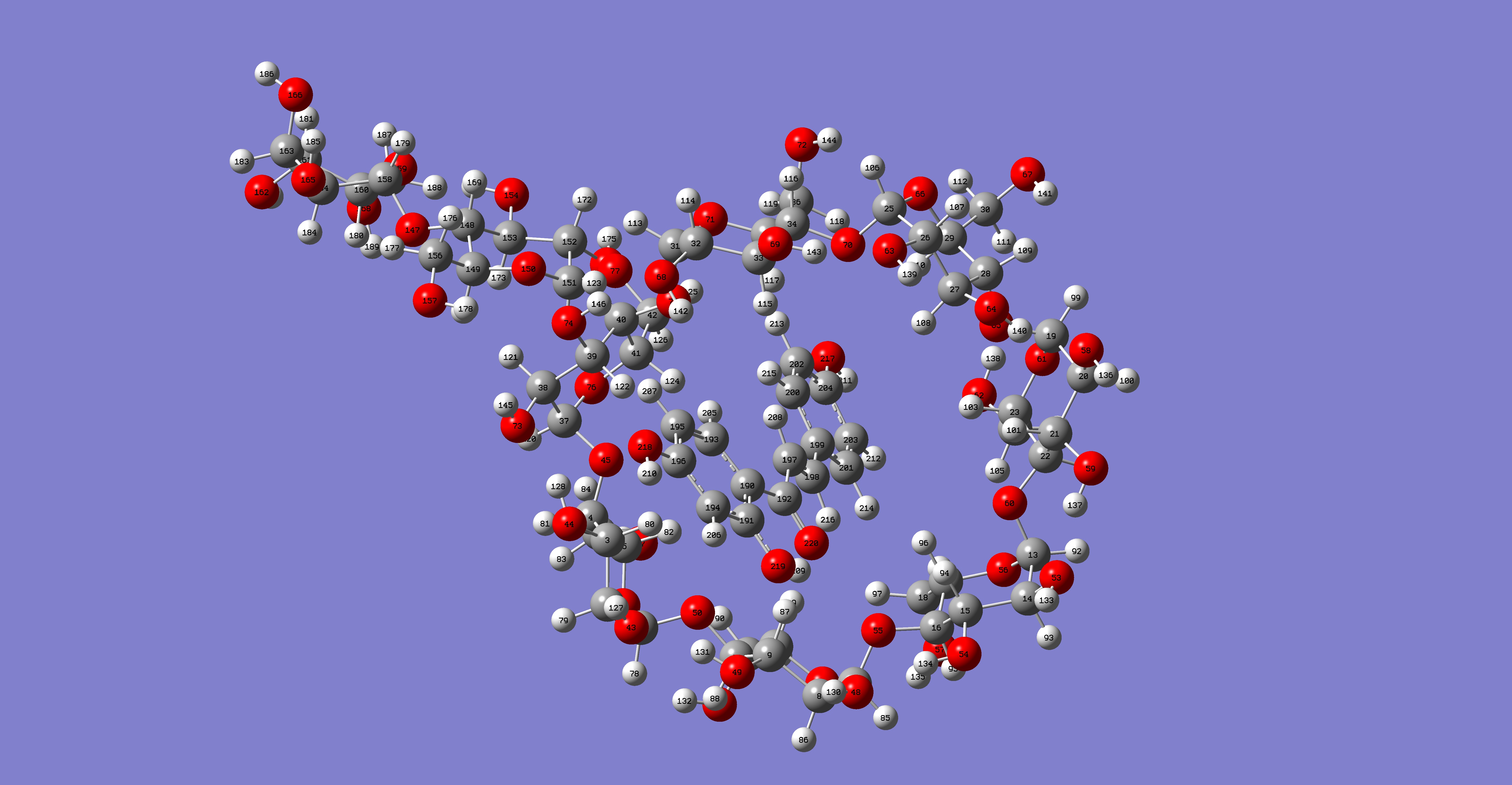

Supplement: Supplementary File 1 [file ijms-16-17999-s001.zip › Supplementary File/SFile2_optimzed_structure.jpg]
